# Aptamer-Conjugated Magnetic Nanoparticles Integrated with SERS for Multiplex Salmonella Detection

**DOI:** 10.3390/bios15070464

**Published:** 2025-07-19

**Authors:** Fan Sun, Kun Pang, Keke Yang, Li Zheng, Mengmeng Wang, Yufeng Wang, Qiang Chen, Zihong Ye, Pei Liang, Xiaoping Yu

**Affiliations:** 1 Key Laboratory of Microbiological Metrology, Measurement and Bio-Product Quality Security, State Administration for Market Regulation, Zhejiang Provincial Key Laboratory of Biometrology and Inspection and Quarantine, College of Life Sciences, China Jiliang University, Hangzhou 310018, China; s23090710042@cjlu.edu.cn (F.S.); pangk@cjlu.edu.cn (K.P.); p24091055074@cjlu.edu.cn (K.Y.); zhye@cjlu.edu.cn (Z.Y.); 2 College of Optical and Electronic Technology, China Jiliang University, Hangzhou 310018, China; s23040809016@cjlu.edu (L.Z.); p23040854112@cjlu.edu.cn (M.W.); plianghust@cjlu.edu.cn (P.L.); 3 College of Metrology and Measurement Engineering, China Jiliang University, Hangzhou 310018, China; chenqiang_cjlu@cjlu.edu.cn

**Keywords:** *Salmonella*, SERS, aptamer, real sample, specific detection

## Abstract

*Salmonella* is a rapidly spreading and widespread zoonotic infectious disease that poses a serious threat to the safety of both poultry and human lives. Therefore, the timely detection of *Salmonella* in foods and animals has become an urgent need for food safety. This work describes the construction of an aptamer-based sensor for *Salmonella* detection, using Fe_3_O_4_ magnetic beads and Ag@Au core–shell nanoparticles-embedded 4-mercaptobenzoic acid (4MBA). Leveraging the high affinity between biotin and streptavidin, aptamers were conjugated to Fe_3_O_4_ magnetic beads. These beads were then combined with Ag@4MBA@Au nanoparticles functionalized with complementary aptamers through hydrogen bonding and π-π stacking interactions, yielding a SERS-based aptamer sensor with optimized Raman signals from 4MBA. When target bacteria are present, aptamer-conjugated magnetic beads exhibit preferential binding to the bacteria, leading to a decrease in the surface-enhanced Raman scattering (SERS) signal. And it was used for the detection of five different serotypes of *Salmonella*, respectively, and the results showed that the aptamer sensor exhibited a good linear relationship between the concentration range of 10^2^–10^8^ CFU/mL and LOD is 35.51 CFU/mL. The SERS aptasensor was utilized for the detection of spiked authentic samples with recoveries between 94.0 and 100.4%, which proved the usability of the method and helped to achieve food safety detection.

## 1. Introduction

*Salmonella* is renowned for its extensive natural distribution and potent infectivity [1], capable of invading a diverse range of organisms, including mammals, amphibians, reptiles, birds, and arthropods [2]. For humans, contaminated foods such as poultry meat, eggs, milk, beef, and water sources are the main routes of *Salmonella* infection [3]. As an important pathogen of foodborne diseases, *Salmonella*, especially the subspecies of *Salmonella* enterica, poses a serious threat to human health [4]. It can cause a series of serious diseases such as typhoid fever, paratyphoid fever, gastroenteritis, septicemia, and local infections. Every year, it causes hundreds of millions of infections and hundreds of thousands of deaths worldwide [5,6]. Given the serious threat of *Salmonella* to food safety and human health, it is particularly important to strengthen the monitoring, prevention, and control of *Salmonella* [7]. Key measures to effectively control *Salmonella* contamination and safeguard human health include improving the sensitivity and accuracy of *Salmonella* detection, enhancing the hygienic management of breeding, slaughtering, processing, and retailing links, as well as increasing the public’s awareness of food safety and self-protection awareness [8].

Currently, the main detection methods for *Salmonella* are as follows: the plate colony counting (cultivation) method based on the differences in the physical and chemical properties of bacteria [9]; the enzyme-linked immunosorbent assay (ELISA) based on the principle of antigen–antibody immune reaction [10]; and the polymerase chain reaction (PCR) based on DNA amplification technology, among which PCR includes various types such as conventional PCR and quantitative PCR. The plate colony cultivation method is the most classic and widely used method for *Salmonella* detection [10]. However, this method has the disadvantages of high labor intensity and a long detection cycle. Generally, the complete detection process takes at least 3–5 days, which makes it impractical for on-site rapid detection scenarios [11]. Nevertheless, ELISA is a multiphase detection technology, and the multiple washing steps in the operation process increase the risk of cross-contamination, which may lead to the occurrence of false positive results [12]. In addition, in terms of detection sensitivity, ELISA has certain limitations, and its low detection limit is usually 10^4^ CFU/mL. The PCR method has the outstanding advantages of high detection sensitivity and short detection time [13]. However, this method has strict requirements for experimental operations, relies on a complex and elaborate DNA extraction process, and requires the operators to have high professional and technical literacy as well as experimental operation skills. Therefore, in order to prevent the spread of *Salmonella* diseases, ensure food safety, and thus protect public health, there is an urgent need for more rapid detection methods for real-time and in situ detection of *Salmonella*.

Surface-enhanced Raman scattering (SERS) technology, with its unique advantages such as high sensitivity, molecular fingerprint recognition ability, surface specificity, wide compatibility, non-destructive detection characteristics, versatility, and rapid detection ability, has become an indispensable analytical tool in the fields of chemistry, biology, materials science, etc. [14]. The nanostructures on the surface of fiber-optic SERS probes enhance Raman signals, enabling rapid detection of unlabeled live *Escherichia coli* with detection times as short as 2.5 milliseconds to 1 s. This provides a new effective method for the rapid detection of bacteria [15]. The study found that mixing bacteria with conventional citrate-reduced silver colloids and drying the suspension can generate highly reproducible surface-enhanced Raman spectra (SERS). These signals originate from intracellular components released during the disruption of bacterial structures in the sample preparation process [16].

Typical surface-enhanced Raman scattering (SERS) probes mainly consist of four parts: a metal nanoparticle substrate, a Raman reporter molecule, a protective shell, and a targeting molecule [17]. Based on biological recognition elements such as antigen/antibody, aptamer [18] (Aptamer, Apt), enzyme catalytic system, whole-cell sensor, molecularly imprinted polymer (MIPs), and phage display technology, combined with signal conversion mechanisms such as optical, electrochemical [19], ultrasensitive detection platforms with a detection limit reaching the level of 1–10 CFU/mL and a detection time shortened to 2–4 h have been developed. Among them, aptamer, as a new type of recognition element, is a short, single-stranded RNA or DNA molecule obtained through the systematic evolution of ligands by exponential enrichment (SELEX) technology and usually has a stable three-dimensional structure [20]. Compared with traditional protein antibodies, aptamers have significant advantages, including high binding affinity, excellent selectivity, easy chemical synthesis and modification, small molecular size, good stability, low batch-to-batch variation, and low immunogenicity [20,21]. As an efficient target molecule affinity reagent, aptamers have received widespread attention in fields such as affinity separation [22], biomarker discovery [23], food safety detection [24], and environmental monitoring [25]. In particular, the analytical methods developed, based on aptamer technology, have made remarkable progress in scientific research and practical applications due to their high sensitivity and specificity [26,27,28].

This work introduced a Ag@4MBA@Au-Fe_3_O_4_ aptamer sensor for ultrasensitive surface-enhanced Raman scattering (SERS) detection of *Salmonella*. Firstly, due to the high affinity between streptavidin on the magnetic bead Fe_3_O_4_ and biotin, a stable non-covalent bond was formed with the aptamer. Subsequently, the Raman reporter molecule 4-mercaptobenzoic acid (4MBA) was embedded in the silver–gold core–shell structure (Ag@Au) nanoparticles. The complementary sequence (c-Apt) of the aptamer was modified onto the surface of Ag@4MBA@Au nanoparticles through Au-S covalent bonds. Finally, through hydrogen bond interactions and the π-π stacking effect of Fe_3_O_4_, stable binding between Fe_3_O_4_-Apt and Ag@4MBA@Au nanoparticles was achieved, completing the construction of the SERS aptamer sensor. Upon exposure to *Salmonella*, the aptamer sensor demonstrates a characteristic decrease in Raman signal intensity. This attenuation results from the specific binding between the aptamer’s defined three-dimensional structure (particularly the G-quadruplex core formed by the folding of guanine-rich (G-rich) regions) and specific epitopes on the surface of *Salmonella* (commonly localized to distinct domains of its outer membrane proteins or lipopolysaccharides), which triggers competitive displacement of the 4MBA-labeled complementary DNA strand. As a result, disruption of the SERS-active ternary complex (Ag@4MBA@Au-c-Apt -aptamer NPs) reduces electromagnetic enhancement at plasmonic hotspots, leading to quantifiable signal quenching. The experimental results showed that the sensor had good stability and exhibited broad-spectrum, high specificity in the detection of *Salmonella*.

## 2. Experimental Section

### 2.1. Materials and Apparatus

Sodium sulfite (Na_2_SO_3_), sodium hydroxide (NaOH), 4-mercaptobenzoic acid (4MBA), polyvinylpyrrolidone (PVP), silver nitrate (AgNO_3_), and ascorbic acid (AA) were obtained from Aladdin Reagent Co., Ltd. (Shanghai, China). Ferric oxide (Fe_3_O_4_) and Gold (III) chloride trihydrate (HAuCl_4_⋅3H_2_O) were purchased from Sinopharm Chemical Reagent Co., Ltd. (Shanghai, China). Ultrapure water (resistivity > 18.2 MΩ cm^−1^) was puriffed using the UPR-II-5/10 T benchtop ultrapure water system (ULUPURE).

For the sequence S11, following the design principle of standard primers, the SH-labeled forward primer was designed as (5′-SH-AAGGGCTGGCTGGGATGGA-3′), and the biotin-labeled reverse primer was designed as (5′-AGTGGGGTCCGTGGAGTGA-Bio-3′) [29,30]. Finally, aptamers were customized by Qingke Biotech Co., Ltd. (Beijing, China).

Transmission electron microscopy (TEM) images were acquired using a JEOL JEM-2010 TEM instrument, while scanning electron microscopy (SEM) images were obtained with an SU8010 instrument. UV-Vis absorption spectra were recorded using a dual-beam UV spectrophotometer model U-3310. SERS spectra were collected utilizing a confocal microscope Raman spectrometer system (Horiba, LabRAM HR) equipped with a 785 nm laser excitation source.

### 2.2. Preparation of Ag@4MBA@Au NPs

Silver nanoparticles were synthesized by following a procedure developed in a previous report [31]. Briefly, a mixture of 0.017 g AgNO_3_ and 100 mL deionized water was put into the flask, a condenser was inserted and fixed, and the flask was heated in an 112 °C oil bath for 5 min (liquid level above oil). Then, 2 mL of 1% sodium citrate solution was quickly added under strong magnetic stirring. The solution changed from colorless to yellow, then to stable yellowish-green. After room-temperature cooling, the Ag nanoparticle solution was stored at 4 °C.

In a 25 mL beaker, 10 mL of Ag NPs and 200 μL of 1 mM 4MBA solution were mixed and magnetically stirred at 600 rpm for 2 h at 25 °C. The solution was centrifuged at 8000 rpm for 15 min, and the precipitate was redispersed in 2 mL ultrapure water to obtain Ag@4MBA NPs.

About 2 mL of the Ag@4MBA solution was transferred to a beaker with a stir bar. After adding 1 mL of 1% PVP and stirring for 5 min, 200 μL of 10 mM AA was added and stirred for 15 min. Au growth solution (22 μL of 1% HAuCl_4_, 240 μL of 0.2 M NaOH, 3 mL of 0.01 M of Na_2_SO_3_) were added dropwise at a constant speed using a pipette and stirred for 30 min to avoid nanoparticle aggregation and uneven gold–shell coating. The solution color changed from yellowish-green to orange-yellow. Finally, the product was centrifuged at 8500 rpm for 15 min, and the precipitate was redispersed in 2 mL ultrapure water. The obtained Ag@4MBA@Au NPs were stored at 4 °C.

### 2.3. Synthesis and Optimization of Ag@4-MBA@Au-Fe_3_O_4_ Aptamer Probes

A total of 40 μL of activated SH-complementary aptamer with a concentration of 10 μM was added to 1 mL of Ag@4MBA@Au NPs solution. The mixture was incubated in an ice bath at −20 °C for 2 h, then centrifuged at 8000 rpm for 10 min to remove the unbound complementary aptamer. After washing with deionized water, 1 mL of deionized water was added to obtain c-Apt-modified Ag@4MBA@Au NPs.

Simultaneously, 40 μL of activated BIO-Apt (10 μM) was added to 20 μL of 10 mg/mL Fe_3_O_4_. The mixture was incubated in an ice bath at −20 °C for 2 h and then centrifuged at 8000 rpm for 10 min to remove excess BIO-Apt. The product was washed with deionized water, collected using a magnet, and dispersed in 1 mL of deionized water to obtain aptamer-modified BIO-Fe_3_O_4_.

A total of 900 μL of Fe_3_O_4_—Apt and 600 μL of complementary aptamer-Ag@4MBA@Au NPs were mixed, gently shaken and incubated in a digital oscillator for 1 h. Subsequently, the mixture was subjected to magnetic separation, washed, and then redispersed in deionized water of the original volume. Thus, Ag@4MBA@Ag-Fe_3_O_4_ magnetic composite nanoparticles were obtained and served as SERS aptamer sensors.

### 2.4. Optimization of Experimental Conditions

In this experiment, the amount of Ag@4MBA@Au NPs was kept constant while the amount of c-Apt added was varied to determine the optimal addition amount. Specifically, different volumes of complementary aptamer (20 μL, 40 μL, 60 μL, 80 μL) were added to 100 μL of Ag@4MBA@Au NPs. After ice-bathing, the mixtures were combined with aptamer-modified Fe_3_O_4_ at a ratio of 2:3, gently shaken on a digital shaker for 1 h, and then subjected to washing and magnetic separation before SERS detection. The optimal c-Apt addition amount was determined based on the intensity of the SERS signals.

To save experimental materials, the amount of Fe_3_O_4_ was fixed while the amount of Apt added was changed to find the optimal addition amount. Specifically, different volumes of Apt (20 μL, 40 μL, 60 μL, 80 μL) were added to 20 μL of Fe_3_O_4_. After ice-bathing, the mixtures were combined with complementary aptamer-modified Au@4MBA@Ag at a ratio of 3:2, gently shaken on a digital shaker for 1 h, and then underwent washing and magnetic separation followed by SERS detection. The optimal aptamer addition amount was determined according to the SERS signal intensity.

For the aptamer sensor, its storage time has a crucial impact on the experimental results. Therefore, a series of experiments were conducted to study the effect of storage time on SERS signals. SERS detection was performed on aptamer sensors freshly prepared (0 days) and they were stored for 5 days, 10 days, 15 days, 20 days, and 25 days. To verify the effectiveness of the aptamer sensor, *Salmonella* with concentrations ranging from 10^1^ CFU/mL to 10^9^ CFU/mL were added to each test.

### 2.5. Activation Culture and Enumeration of Salmonella

The original strains of *Salmonella* were inoculated into Luria–Bertani medium (LB liquid medium) under sterile conditions and placed at 37 °C for 12 h with shaking to activate and increase bacteria. Approximately 25 mL of *Salmonella*-like solution was pipetted and placed in a sterile Erlenmeyer flask containing 225 mL of normal saline, and it was shaken well to make the original *Salmonella* solution. Under sterile conditions, 100 μL of the original bacterial solution was slowly injected into a sterilized centrifuge tube filled with 900 μL of sterilized normal saline and shaken to mix it thoroughly to make a 1:10 sample bacterial solution. The previous operation steps were repeated, ten-fold concentration gradient dilution was carried out, and strict sterility was ensured during the operation. Three plates with dilution factors of 10^−5^~10^−7^ were selected and counted, and 100 μL of *Salmonella* solution was injected into the LB solid plate culture base to coat the plate, and each concentration was coated in parallel with three plates. They were incubated at 37 °C, observed and counted, and the dilution factor and the corresponding number of colonies were recorded. The colony-forming units (CFU) are used to represent the total number of colonies. When counting, the number of colonies between 30 and 300 CFUs and the colony growth plate without spread should be selected, and the concentration of the original bacterial solution can be calculated according to the dilution factor and the number of colonies [32].

### 2.6. SERS Detection

For the SERS detection of *Salmonella*, first, 50 μL of *Salmonella* standard solutions at different concentrations (1 × 10^9^ CFU/mL, 1 × 10^8^ CFU/mL, 1 × 10^7^ CFU/mL, 1 × 10^6^ CFU/mL, 1 × 10^5^ CFU/mL, 1 × 10^4^ CFU/mL, 1 × 10^3^ CFU/mL, 1 × 10^2^ CFU/mL, 1 × 10^1^ CFU/mL) were, respectively, added to 100 μL of the prepared aptamer sensor solution. The mixtures were incubated at 37 °C for 1 h. Due to the binding between the aptamer and *Salmonella*, part of the Ag@4MBA@Au-complementary aptamer detached from Fe_3_O_4_—Apt. After centrifugation and washing with deionized water, the detached complementary aptamer-Ag@4MBA@Au was completely removed. Then, the obtained product was fully redispersed in 10 μL of PBS buffer, dropped onto a silicon wafer, and dried under vacuum.

The SERS spectra were obtained using a confocal Raman microscope spectrometer. The acquisition parameters were set as follows: laser power of 200 mW, integration time of 5 s, number of integrations was 1, and the scanning range was 800 cm^−1^–1800 cm^−1^. For each sample, five repeated SERS signals were collected, and the average value was taken as the valid result. The peak value of 4MBA at 1078 cm^−1^ was recorded for subsequent analysis of *Salmonella*.

## 3. Results and Discussion

### 3.1. Detection Principle of the Aptamer Sensor

**Figure 1:**
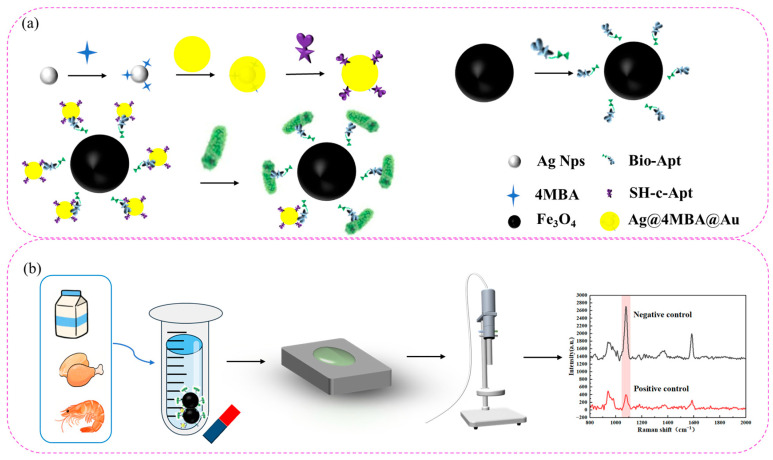
(**a**) Schematic illustration of the fabrication and detection principles of the aptamer-based biosensor; (**b**) procedure of the aptamer probe for detecting real samples.

Figure 1 illustrates the detection principle of the SERS aptamer sensor for detecting *Salmonella*, which is based on aptamers, SERS, Fe_3_O_4_, and Ag@4MBA@Au nanoparticles. Firstly, the Raman reporter molecule 4MBA is embedded in the Ag@Au nanoparticles. This core–shell structure composed of heterogeneous metals not only significantly enhances the SERS signal but also protects the embedded Raman signal from external interference, greatly enhancing and stabilizing the SERS signal. Magnetic beads bind to aptamers through the high affinity between streptavidin and biotin to form stable non-covalent bonds. SH-complementary aptamer binds to Ag@4MBA@Au nanoparticles via Au-S covalent bonds. Subsequently, Fe_3_O_4_—Apt and complementary aptamer-Ag@4MBA@Au are combined through hydrogen bonds and π-π stacking interactions, thus constructing the SERS aptamer sensor for detecting *Salmonella*. At this point, due to multiple SERS enhancement effects, the aptamer sensor exhibits the strongest SERS signal intensity. Finally, when *Salmonella* is added, because of the high specificity of the aptamer, it preferentially binds to *Salmonella*. This causes Ag@4MBA@Au to detach from Apt-Fe_3_O_4_. Upon exposure to *Salmonella*, the aptamer sensor demonstrates a characteristic decrease in Raman signal intensity. This attenuation arises from the specific binding of aptamers to bacterial surface epitopes, which triggers the competitive displacement of 4MBA-labeled complementary DNA strands. As a result, disruption of the SERS-active ternary complex (aptamer-c-Apt-Ag@4MBA@AuNP) reduces electromagnetic enhancement at plasmonic hotspots, leading to quantifiable signal quenching. The experimental results showed that the sensor had good stability and exhibited broad-spectrum, high specificity in the detection of *Salmonella*. Therefore, based on the inverse relationship between the concentration of *Salmonella* and the SERS intensity, different concentrations of *Salmonella* can be quantitatively determined.

### 3.2. Characterization of the Ag@4MBA@Au-Fe_3_O_4_ Composite Probe

Ag@4MBA@Au NPs, serving as the SERS substrate, combine the advantages of two types of nanoparticles and can provide relatively stable, reliable, and highly amplified Raman signals. However, the thickness of the silver core and the gold shell can affect the SERS activity.

**Figure 2:**
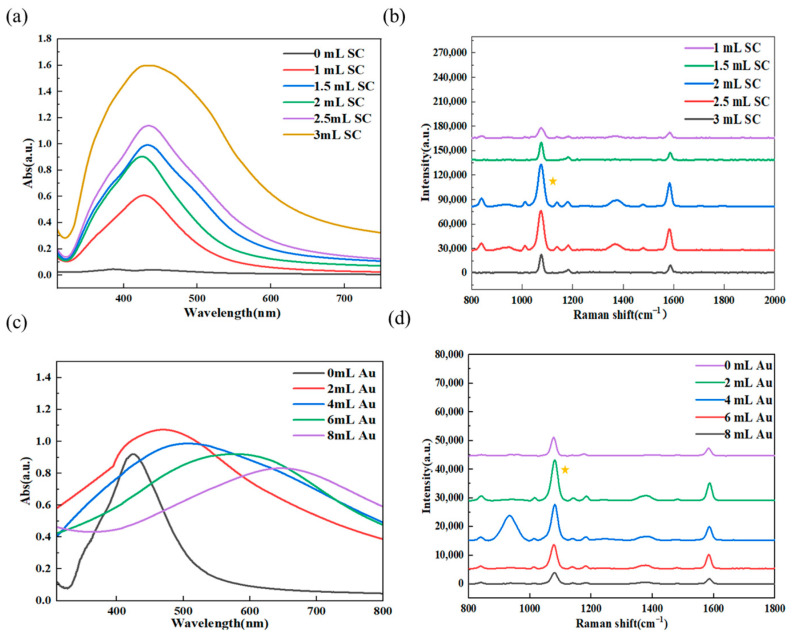
(**a**) UV-Vis spectra with different silver core thicknesses, (**b**) SERS spectra with different silver core thicknesses, (**c**) UV-Vis spectra with different gold shell thicknesses, and (**d**) SERS spectra with different gold shell thicknesses. Small golden stars represent the highest SERS signal.

Raman spectroscopy tests were carried out on Ag@4MBA with different silver core thicknesses. As shown in Figure 2a,b, the experimental results indicate that as the thickness of the silver core increases, the SERS amplification signal is the strongest when 2 mL of 1% sodium citrate solution is added. When the amount of 1% sodium citrate solution exceeds 2 mL, the SERS amplification signal weakens, and the resolution of the characteristic peaks decreases. This is because when the amount of 1% sodium citrate solution exceeds 2 mL, the core size of the nanoparticles becomes too large, causing aggregation and precipitation of Ag@4MBA NPs, which in turn affects SERS activity. Therefore, the optimal addition amount of 1% sodium citrate solution for the silver nanocore is 2 mL.

Raman spectroscopy tests were carried out on Ag@4MBA@Au with different thicknesses of gold nanoparticle shells. As shown in Figure 2c,d, the experimental results revealed that with the increase in the amount of Au growth solution, the absorbance peak of Ag@4MBA@Au gradually shifted to the right, and the SERS amplification signal obtained when 2 mL of Au growth solution was added was the strongest. When the addition amount of Au growth solution exceeded 2 mL, the SERS amplification signal weakened, and the resolution of characteristic peaks decreased. This is because when the addition amount of Au growth solution exceeds 2 mL, resulting in an excessively large shell thickness of the nanoparticles, it will cause the aggregation and precipitation of Ag@4MBA@Au NPs, thereby affecting SERS activity. Therefore, the optimal addition amount of Au growth solution that affects the gold nanoparticle shell is 2 mL. The particle size of the Ag@4MBA@Au structure is approximately 48 nm.

**Figure 3:**
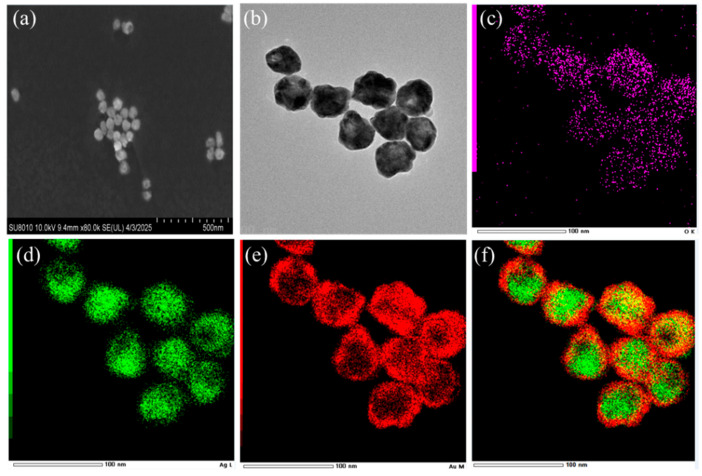
(**a**) The SEM image of Ag@4MBA@Au, (**b**) The TEM image of Ag@4MBA@Au, (**c**) O elemental map, (**d**) Ag elemental map, (**e**) Au elemental map, and (**f**) Ag/Au elemental map from EDS analysis of the Ag@4MBA@Au nanostructure.

The SEM characterization results of Ag@4MBA@Au are shown in Figure 3a,b. It can be clearly observed that the morphology of Ag@4MBA@Au is distinct, and the Ag@4MBA@Au nanoparticles are uniform in size. To further validate the successful preparation of the internal standard-embedded core–shell structure, in this study, a transmission electron microscope (TEM) was used to characterize and analyze the Ag@4MBA@Au NPs. The scanning results for Ag, Au, and S elements are shown in Figure 3c–f. Given that 4MBA contains oxygen (O), the distribution of Au, Ag, and O elements in the EDS maps confirms the successful embedding of 4MBA between the core and shell. This result provides a direct visual basis for revealing the microscopic structure of the nanoparticles and subsequent studies on their properties.

### 3.3. Results of Experimental Condition Optimization

**Figure 4:**
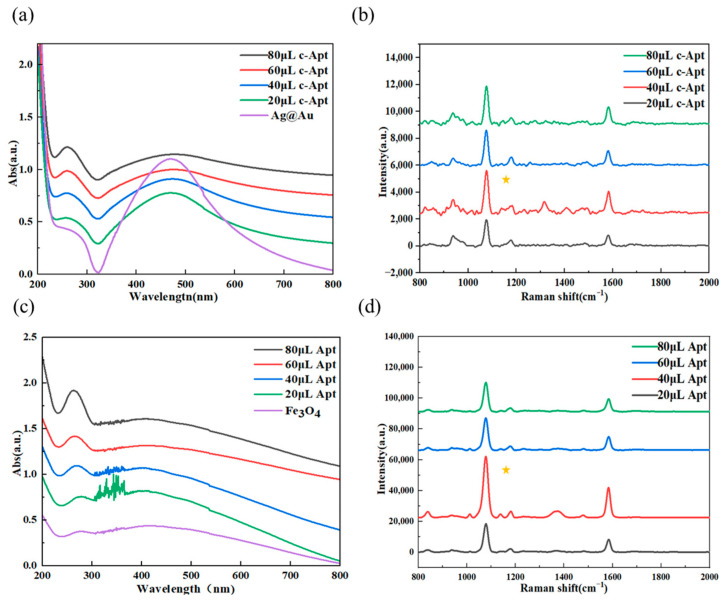
(**a**) UV-Vis spectra with different addition amounts of c-Apt, (**b**) SERS spectra with different addition amounts of c-Apt, (**c**) UV-Vis spectra with different addition amounts of Apt, and (**d**) SERS spectra with different addition amounts of Apt.

In this experiment, to explore the optimal addition amount of c-Apt in the Ag@4MBA@Au-Fe_3_O_4_ aptamer probe complex for obtaining the strongest Raman signal from the complex, the amount of Apt was kept constant at 20 μL while the amount of added c-Apt varied. Firstly, to verify the successful modification of c-Apt on the surface of Ag@4MBA@Au, ultraviolet–visible (UV-Vis) absorption spectroscopy was conducted on the solutions before and after modification. The results are presented in Figure 4a. It can be observed that the peak of Ag@4MBA@Au near 480 nm remained unchanged before and after aptamer modification, indicating that the addition of aptamers did not alter the structure of the nanoparticles. However, a new peak emerged at approximately 260 nm after aptamer modification, corresponding to the classic absorption peak of the aptamer, which confirms the successful modification of aptamers on the surface of Ag@4MBA@Au NPs. This characteristic peak is clear and distinct, providing important spectroscopic evidence for the subsequent research and characterization of the c-Apt’s related properties. The appearance of this absorption peak indicates that the modification process does not damage the basic structure of the c-Apt, and the c-Apt still maintains its inherent nucleic acid absorption characteristics. As shown in Figure 4b, with the increase in the amount of c-Apt, the SERS signal intensity of 4MBA at 1078 cm^−1^ gradually increased. When the amount of c-Apt reached 40 μL, the signal intensity reached its maximum value. Further increases in the c-Apt amount led to insignificant changes or even a slight decrease in the signal intensity. This is because Ag@4MBA@Au—c-Apt almost occupied all the binding sites on Fe_3_O_4_- Apt. With both ensuring the optimal Raman signal intensity and saving reagent costs taken into consideration, 40 μL was selected as the optimal addition amount.

In this experiment, to investigate the optimal addition amount of Apt in the Ag@4MBA@Au-Fe_3_O_4_ aptamer probe complex for achieving the strongest Raman signal of the complex, the amount of c-Apt was maintained at a constant 40 μL while the amount of added Apt was altered. As shown in Figure 4c, the ultraviolet–visible spectroscopy analysis indicates that the chemically modified aptamer exhibits a characteristic absorption peak at 260 nm. As depicted in Figure 4d, as the amount of Apt increased, the SERS signal intensity of 4MBA at 1078 cm^−1^ gradually enhanced. When the amount of Apt reached 40 μL, the signal intensity peaked. After that, with further increases in the Apt amount, the change in the signal intensity became insignificant or even showed a slight decrease. This was because Fe_3_O_4_—Apt almost occupied all the sites on Ag@4MBA@Au—c-Apt. Under the premise of ensuring the best Raman signal intensity and saving reagent costs, 40 μL was chosen as the optimal addition amount.

**Figure 5:**
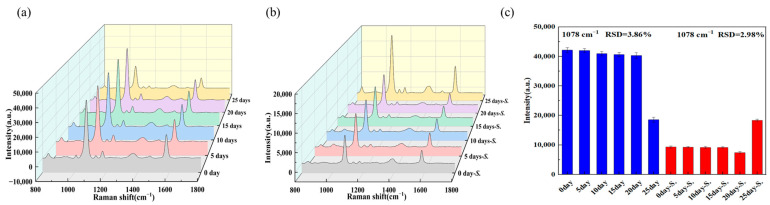
(**a**) SERS spectra of the aptamer probe affected by the storage time, (**b**) SERS spectra of the aptamer probe for detecting 10^8^ CFU/mL influenced by the storage time, (**c**) and RSD (Relative Standard Deviation) diagram of the influence of the storage time on the aptamer probe.

For the aptamer sensor, the storage time has a crucial impact on the experimental results. Thus, a series of experiments were carried out to study the effect of storage time on the SERS signal. SERS detection was performed on aptamer sensors freshly prepared (0 days) and those stored for 5 days, 10 days, 15 days, 20 days, and 25 days. To verify the effectiveness of the aptamer sensor, *Salmonella* suspensions at 10^8^ CFU/mL were spiked into each test system. As shown Figure 5a–c, it can be seen that the aptamer sensors stored for 20 days, 15 days, 10 days, and 5 days maintained good Raman intensities. After adding *Salmonella* typhimurium, the Raman intensity decreased significantly, which proved the effectiveness of the aptamer sensors. For the aptamer sensor stored for 25 days, the Raman intensity decreased slightly, and after adding *Salmonella* typhimurium, there was almost no change in the Raman intensity, indicating that the aptamer sensor stored for 25 days had become ineffective. The instability of the Raman signal from silver core, gold shell (Ag@Au) nanostructures after 20 days arises primarily from the interplay of structural, chemical, and environmental factors. Structurally, the silver core is prone to oxidation or dissolution, leading to degradation of the core–shell architecture—this alters the core dimensions, disrupts the uniformity of the gold shell, and weakens localized surface plasmon resonance (LSPR) coupling. Chemically, the gold shell undergoes surface modifications: adsorption of contaminants blocks “hotspots”, while interdiffusion with the silver core forms alloys, both of which alter plasmonic properties. Additionally, the failure of surface stabilizers causes particle aggregation, reducing dispersion stability, shifting LSPR peaks, and diminishing hotspot density. Environmental fluctuations, such as variations in temperature, humidity, pH, and light exposure, accelerate these processes, further exacerbating structural damage. Therefore, the aptamer sensors prepared in this experiment can be stably used within the first week.

### 3.4. Detection of Salmonella

**Figure 6:**
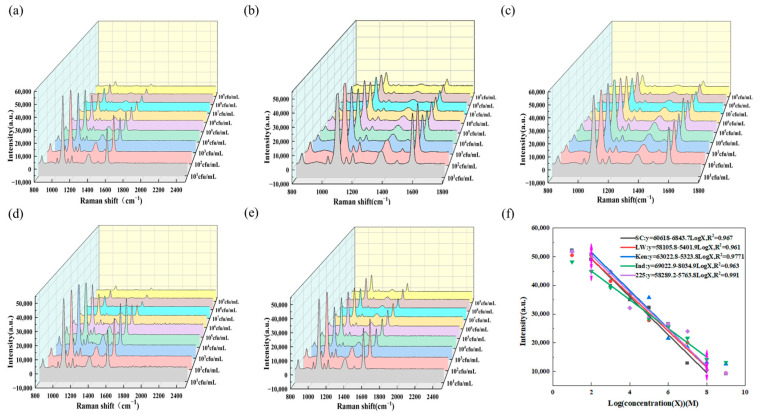
(**a**) SERS spectra of the S11 sequence aptamer probe for detecting *Salmonella* typhimurium at different concentrations. (**b**) SERS spectra of the S11 sequence aptamer probe for detecting *Salmonella* enteritidis at different concentrations. (**c**) SERS spectra of the S11 sequence aptamer probe for detecting *Salmonella* Kentucky at different concentrations. (**d**) SERS spectra of the S11 sequence aptamer probe for detecting *Salmonella* Indiana at different concentrations. (**e**) SERS spectra of the S11 sequence aptamer probe for detecting *Salmonella* 225 at different concentrations. (**f**) Linear fitting diagrams of the S11 sequence aptamer probe for detecting *Salmonella* typhimurium, *Salmonella* enteritidis, *Salmonella* Kentucky, *Salmonella* Indiana, and *Salmonella* 225 at different concentrations.

Under optimal detection conditions, the SERS intensity of the sensor for different concentrations of *Salmonella* was investigated. As shown in Figure 6a–e, the detection results display SERS signals at concentrations ranging from 10^2^ CFU/mL to 10^8^ CFU/mL. With the increase in *Salmonella* concentration, more *Salmonella* bind to aptamers, leading to the release of more Au@4MBA@Ag—c-Apt from the aptamer sensor. In Figure 6a–f, there is a good linear relationship between the SERS intensity at 1078 cm^−1^ and Log(concentration) in the range from 10^2^ CFU/mL to 10^8^ CFU/mL. The linear regression equation for the detection of *Salmonella* typhimurium by the aptamer probe with the S11 sequence is y = −6843.7LogX + 60,618, and the coefficient of determination R^2^ is 0.967. The linear regression equation for the detection of *Salmonella* enteritidis is y = −5401.9LogX + 58,105.8, and R^2^ is 0.961. The linear regression equation for the detection of *Salmonella* Kentucky is y = −5323.8LogX + 63,022.8, and R^2^ is 0.977. The linear regression equation for the detection of *Salmonella* Indiana is y = −8034.9LogX + 69,022.9, and R^2^ is 0.963. The linear regression equation for the detection of *Salmonella* 225-170309038 is y = −5763.8LogX + 58,289.2, and R^2^ is 0.991. We can observe that the linear curve of *Salmonella* Indiana in Figure 6f differs from those of other serotypes. Although the five *Salmonella* serovars share conserved genomic architecture, S. Indiana exhibits attenuated ligand-binding activity attributable to two distinct molecular perturbations: (i) deficient O-antigen polymerization in the lipopolysaccharide (LPS) core oligosaccharide region, and (ii) susceptibility to global regulatory anomalies. These factors collectively manifest as a statistically significant deviation in dose–response slope (p* < 0.01) relative to the other four serovars.

### 3.5. Specificity Detection

**Figure 7:**
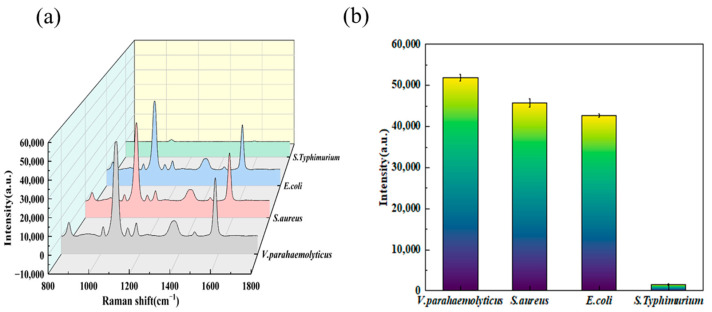
(**a**) SERS spectra of the S11 sequence aptamer probe for detecting *Staphylococcus aureus*, *Vibrio parahaemolyticus*, *Escherichia coli*, and *Salmonella*. (**b**) RSD (Relative Standard Deviation) diagrams of the S11 sequence aptamer probe for detecting *Staphylococcus aureus*, *Vibrio parahaemolyticus*, *Escherichia coli*, and *Salmonella*.

To verify the specificity of the two aptamer sensors, three foodborne pathogenic bacteria were selected for comparison: *Escherichia coli* (*E. coli*), *Vibrio parahaemolyticus*, and *Staphylococcus aureus* (*S. aureus*). These bacteria were added to the aptamer sensors at concentrations ranging from 10^1^ to 10^9^ CFU/mL to study the specificity of the *Salmonella* aptamer sensors. Figure 7a,b shows the SERS spectra of the S11 aptamer sensor detecting the three bacteria. When the same-concentration bacterial solutions were added, the SERS signals of these three analytes remained stable. This result clearly indicates that the SERS-based aptamer sensor exhibits high specificity in detecting *Salmonella*. This high specificity can be attributed to the high specificity of the *Salmonella* aptamer, enabling the aptamer sensor to distinguish *Salmonella* from other substances at lower concentrations.

### 3.6. Detection of Actual Samples

The preparation of milk samples was carried out according to the procedure developed in previous reports [33]. Briefly, 1 mL of fresh pure milk was centrifuged at 12,000 rpm/min for 5 min. The supernatant was discarded to remove the fat in the milk. Then, 1 mL of sterile water was added, and the mixture was vortexed and centrifuged at 12,000 rpm for 2 min. The supernatant was discarded, and this process was repeated twice. After that, 1 mL of sterile water was added again, and the mixture was centrifuged at 12,000 rpm for 5 min. Afterwards, 1 mL of the supernatant was collected and diluted to 10 mL with ultrapure water to obtain the milk sample solution. Using the milk sample solution as the matrix, 200 μL of *Salmonella* was added, and the mixture was incubated at 37 °C for 6 h. The optical density (OD) was measured, and the solution was diluted to prepare a concentration gradient ranging from 1 × 10^1^ to 1 × 10^9^ CFU/mL for SERS detection to obtain the standard curve.

In this experiment, the determination of *Salmonella* in commercially available chicken meat (purchased from Vanguard Supermarket) was selected as an example. Under sterile conditions, 5 g of fresh chilled chicken breast was mixed with 45 mL of sterilized nutrient broth medium. The mixture was aseptically crushed and equally divided into 4 parts. Each part was centrifuged at 3000 rpm for 5 min in a centrifuge, and the suspension was filtered through a 0.45 μm filter membrane. The filtered broth was stored at −20 °C for later use. Using the filtered broth sample solution as the matrix, 200 μL of *Salmonella* was added, and the mixture was incubated at 37 °C for 6 h. The OD was measured, and the solution was diluted to prepare a concentration gradient from 1 × 10^1^ to 1 × 10^9^ CFU/mL for SERS detection to obtain the standard curve [34].

Fresh shrimp were purchased from a local supermarket and placed in a sealed box with ice packs until the experiment. Approximately 20 g of shrimp samples were aseptically crushed and homogenized in 80 mL of alkaline peptone containing 3% sodium chloride for 30 min. After standing for 1 h to precipitate large aggregates and substrates, the supernatant was further collected for use. Using the supernatant as the matrix, 200 μL of *Salmonella* was added, and the mixture was incubated at 37 °C for 6 h. The OD was measured, and the solution was diluted to prepare a concentration gradient from 1 × 10^1^ to 1 × 10^9^ CFU/mL for SERS detection to obtain the standard curve.

**Figure 8:**
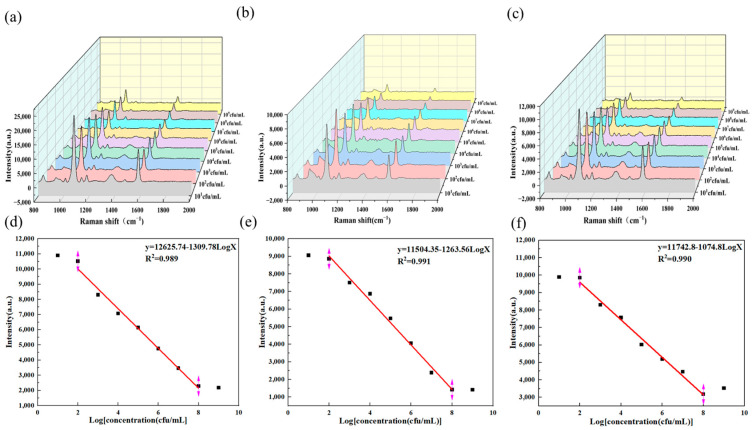
(**a**) SERS spectra of the S11 sequence aptamer probe for detecting *Salmonella* typhimurium at different concentrations in milk samples. (**b**) SERS spectra of the S11 sequence aptamer probe for detecting *Salmonella* typhimurium at different concentrations in chicken samples. (**c**) SERS spectra of the S11 sequence aptamer probe for detecting *Salmonella* typhimurium at different concentrations in shrimp samples. (**d**) Linear fitting diagrams of the S11 sequence aptamer probe for detecting *Salmonella* typhimurium at different concentrations in milk samples. (**e**) Linear fitting diagrams of the S11 sequence aptamer probe for detecting *Salmonella* typhimurium at different concentrations in chicken samples. (**f**) Linear fitting diagrams of the S11 sequence aptamer probe for detecting *Salmonella* typhimurium at different concentrations in shrimp samples.

Figure 8a–f shows the Raman spectra at different concentrations. According to the analysis of the SERS sensor, when *Salmonella* was added to milk, chicken, and shrimp, the linear fitting of the Raman intensity at 1076 cm^−1^ was also recorded. The linear regression equations for the detection of *Salmonella* in milk, chicken, and shrimp using the aptamer probe with the S11 sequence are all y = −1309.78LogX + 12,625.74 (R^2^ = 0.989), y = −1263.56LogX + 11,504.35 (R^2^ = 0.991), and y = −1074.80LogX + 11,742.80 (R^2^ = 0.990)

A total of 50 samples were randomly selected from the above-mentioned actual samples as blind samples, including 20 milk samples, 15 chicken samples, and 15 shrimp samples, with each category forming a group. For each sample in each group, 10^3^ CFU/mL of *Salmonella* typhimurium or *Vibrio parahaemolyticus* was randomly added, or no bacteria were added. A blank control group was set up. The bacterial suspension was centrifuged at 3000 rpm for 5 min, and then the supernatant was discarded. Approximately 1 mL of the sample solution was added to each sample to prepare differently spiked samples. The SERS method was applied to measure these spiked samples. The blind-testing mode of a portable surface-enhanced Raman spectroscopy rapid-detection product was adopted, and the principle of separating sample preparation from detection was implemented. That is, the operators responsible for sample preparation of the rapid-detection product and those for detection using the reference method were independent of each other to ensure the objectivity and reliability of the detection results.

**Figure 9:**
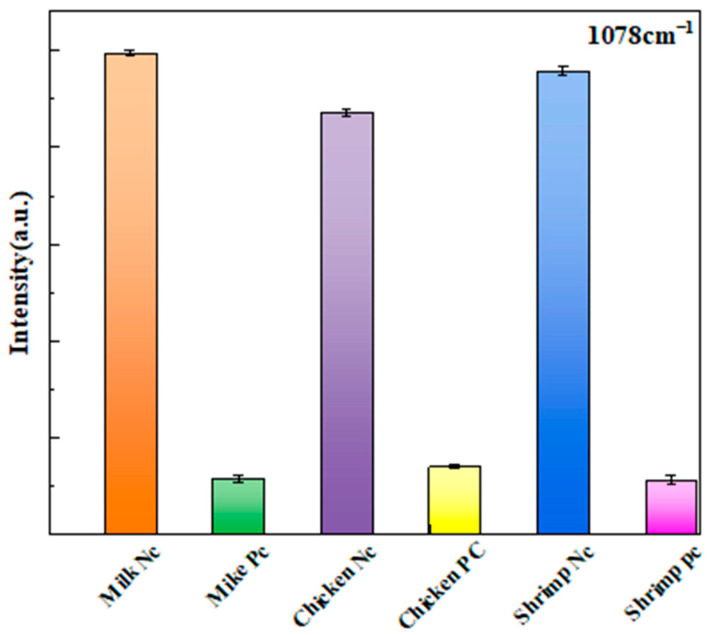
SERS detection was conducted at 1078 cm^−1^ on negative and positive samples of milk, chicken, and shrimp.

As shown in Figure 9 and the Table 1 below, the aptamer probe sensor can accurately detect the presence of *Salmonella*. Additionally, the recovery rates of *Salmonella* in different matrices were determined to be between 94.0% and 100.4%.

## 4. Summary and Outlook

This study presents the development of an aptamer sensor incorporating magnetic materials and Ag@4MBA@Au composite nanoparticles, which integrates specific aptamers with surface-enhanced Raman scattering (SERS) technology to enable efficient detection of *Salmonella* through specific aptamer–bacteria binding. The sensor employs an innovative competitive mechanism: upon the presence of *Salmonella*, the bacteria compete with Ag@4MBA@Au nanoparticles for binding sites on magnetic materials, inducing detachment of beacon molecule (4MBA)-conjugated nanomaterials and subsequent reduction in detection signals. The nanocomposites were systematically optimized and characterized in terms of morphology, structure, and SERS performance. The developed *Salmonella*-specific aptamer sensor exhibits excellent stability, high detection specificity, a broad linear range of 10^2^–10^8^ CFU/mL under optimized conditions, and spiked recovery rates of 94.0–100.4% in real-world samples, validating the method’s feasibility and practical applicability. Detection and analysis of actual samples further confirm the sensor’s capability to effectively discriminate *Salmonella* from other foodborne pathogens.

## Figures and Tables

**Table 1:** Real sample detection results.

Sample Types	Total	Negative Count	Positive Count	Recovery Rate (%)	Positive Rate (%)
milk	20	9	11	95.6–100.4	100
chicken	15	7	8	94.0–100.1	100
shrimp	15	7	8	94.0–100.0	100

## Data Availability

The complete data of this study can be obtained upon request from the corresponding author.
